# Dermatofibrosarcoma protuberans: surgical management of a challenging mesenchymal tumor

**DOI:** 10.1186/s12957-019-1627-3

**Published:** 2019-05-28

**Authors:** Felix Wiesmueller, Abbas Agaimy, Aristotelis Perrakis, Andreas Arkudas, Raymund E. Horch, Robert Grützmann, Nikolaos Vassos

**Affiliations:** 1 Department of Surgery, University Hospital Erlangen, Friedrich-Alexander-University of Erlangen-Nuremberg (FAU), Krankenhausstraße 12, 91054 Erlangen, Germany; 2 Institute of Pathology, University Hospital Erlangen, Friedrich-Alexander-University of Erlangen-Nuremberg (FAU), Erlangen, Germany; 3 Department of Plastic and Hand Surgery, University Hospital Erlangen, Friedrich-Alexander-University of Erlangen-Nuremberg (FAU), Erlangen, Germany; 4 Department of Surgery, University Hospital Magdeburg, Magdeburg, Germany; 5 Division of Surgical Oncology and Thoracic Surgery, Department of Surgery, University Medical Center Mannheim, University of Heidelberg, Mannheim, Germany

**Keywords:** Dermatofibrosarcoma protuberans, DFSP, Cutaneous lesions, Mesenchymal tumor, Wallace rule of 9s

## Abstract

**Introduction:**

Dermatofibrosarcoma protuberans (DFSP) is a rare, low-grade malignant mesenchymal tumor of the soft tissue, characterized by slow infiltrative growth and common local recurrence, with rare distant metastases.

**Patients and methods:**

We present a retrospective study of nineteen patients who were diagnosed with DFSP and operated at our institution in > 10-year period. We examined the clinicopathological parameters with special emphasis on the margin status regarding the clinical outcome and the follow-up.

**Results:**

A total of eight cases underwent re-excision at our institution following primary excision or incisional biopsy performed at a different institution. Seven cases received excision after incisional biopsy at our institution. Four patients developed recurrent disease following primary excision with histological R0 margins at other institutions and received re-excision at our institution. All excisions at our institution resulted in R0 margins with no recurrence recorded at last follow-up (6 to 175; mean 84 months). The mean margin for those who received resection at our institution was 1.67 cm. Negative margins upon primary excision were achieved using a mean margin width of 2.04 cm. Most common tumor localization was the trunk (10 cases).

**Conclusion:**

Awareness of this rare entity is important for a prompt diagnosis and a proper management of the disease. The greatest clinical challenge in the management of DFSP is achieving local control. Complete excision of the tumor with surgical margin widths of at least 2 cm is recommended.

## Introduction

Dermatofibrosarcoma protuberans (DFSP) is a rare low-grade soft tissue sarcoma accounting for approximately 1–2% of sarcoma with an annual incidence rate of 0.8–4.1 cases per million persons per year [1, 2]. This dermal tumor was first described by Taylor [3], clinically was classified by Darier and Ferrand [4] and later named by Hoffman [5]. Although congenital cases have been described, DFSP typically arises in the third decade of life [6–8]. This rare tumor is clinically characterized by an indolent clinical course since it grows slowly, similar to nodules that appear as hypertrophic scars or benign soft tissue tumors without any specific symptoms. Locally however, DFSP exhibits an aggressive behavior, spreading within the dermis, subcutaneous tissue, and ultimately into muscles, with finger-like extensions. Hematogenous or lymphatic dissemination is very rare in conventional DFSP.

Approximately 10–15% of all DFSPs shows transition to a spindle cell sarcoma closely similar to adult fibrosarcoma, frequently associated with increased mitotic activity and variable loss of CD34 expression. Fibrosarcomatous dermatofibrosarcoma protuberans (FS-DFSP) is considered to be intermediate-grade neoplasm with a slightly increased risk of distant metastasis compared to ordinary DFSP [2, 9].

The greatest clinical challenge in the management of DFSP is achieving local control. Because DFSP arises in the dermis and invades radially through preexisting collagen bundles and deeply along connective tissue septae, its extent of invasion is often difficult to clinically appreciate, and thus determining the appropriate margin width is frequently challenging.

The goal of this retrospective study is to provide the data of patients treated for DFSP at our institution and to expand the clinicopathological characteristics of this unusual neoplasm for optimizing therapeutic strategies. We also hypothesize that tissue found on body surface areas (BSA) other than the trunk might be equally likely or more likely to foster growth of DFSP. Awareness of this rare entity is important for a prompt diagnosis and a proper management of the disease, preventing over- and undertreatment of this low to intermediate-grade malignancy. Therefore we want to highlight this tumor that should be considered in the differential diagnosis of cutaneous lesions.

## Patients and methods

A retrospective chart review was compiled (Table 1). Patients operated with a diagnosis of primary DFSP between 2002 and 2016 were included in the chart. Patient data were collected from medical records of the Department of General and Visceral Surgery, University Hospital Erlangen, Germany. Data included demographics (age, gender, history), tumor presentation and characteristics (location, size), treatment modality and closure type, histopathological report, and disease evolution (location and time of recurrence, follow-up).

**Table 1:** Clinicopathological parameters of patients with DFSP

Patient number	Group (A–C)	Gender (M/F)	Diagnosis	Age at diagnosis (years)	Localization	Presentation	Tumor specimen diameter (cm)	Resection margins (cm)	Closure type	Neoadjuvant therapy	Histopathology	Final resection status	Local recurrence free survival after R0 resection (months)
1	A	F	DFSP	42	Neck	On normal cutis	1.5	1	Primary	n/a	CD34+, Vimentin+, sm-Actin+	R0	175
2	C	M	DFSP	46	Trunk	On normal cutis	3.1	2	TRAM flap	n/a	CD34+, Actin neg, MIB1 3%	R0	145
3	B	M	DFSP	74	Gluteal	On normal cutis	2.3	2	Primary	n/a	CD34+, MIB1 15%	R0	135
4	A	F	DFSP	70	Trunk	On normal cutis	6.5	1	Primary	Imatinib mesylate	CD34+, MIB1 2%	R0	58
5	A	M	DFSP	34	Trunk	On normal cutis	3.0	1	Primary	n/a	CD34+, vimentin+	R0	113
6	C	F	DFSP	36	Trunk	On normal cutis	1.7	0.5	Pectoralis flap	n/a	CD34+, S100neg., Desmin neg., spindle cells	R0	105
7	B	M	DFSP	33	Hand	On normal cutis	0.3	0.8	Gracilis flap	n/a	CD34+, MAC387+ macrophages, factor VIII+ endothelial cells, MIB1 1%	R0	103
8	B	M	DFSP	54	Trunk	On scar from lipoma excision	2.0	2.5	LICAP flap	n/a	CD34+	R0	95
9	B	F	DFSP with high-grade sarcomatous transfomation	46	Gluteal	On normal cutis	4.6	3	ALT flap	Radiation therapy (60 Gy) and ifosfamide/doxorubicin	CD34+, ß-catenin+, CD99+, MIB1 50%	R0	87
10	B	M	DFSP	46	Trunk	On normal cutis	3.3	3	Primary	n/a	CD34+, PDGFR-alpha+, PDGFR-beta+. MIB1 5–10%	R0	88
11	A	F	DFSP	50	Trunk	On normal cutis	2.0	1	Primary	n/a	CD34+, MIB 2%	R0	84
12	B	F	DFSP	89	Upper extremity	On normal cutis	1.7	1	Perforator flap	n/a	CD34+	R0	6
13	A	M	DFSP	46	Inguinal	On prior scar	6.5	2	Primary	n/a	CD34+, PDGFR-beta+	R0	63
14	C	F	DFSP	44	Trunk	On irritated cutis	3.5	1	Skin graft	n/a	CD34+, PDGFR-beta+, p16+, MIB 1–5%	R0	62
15	A	F	DFSP	43	Trunk	On normal cutis	3.0	2	Perforator flap	n/a	CD34+, MIB 10%	R0	55
16	A	F	DFSP	56	Lower extremity	On normal cutis	1.8	2	ALT flap	n/a	CD34+, CD117+	R0	48
17	B	M	DFSP	81	Gluteal	On normal cutis	2.9	2	Primary	n/a	Exophytic tumor, spindle cells; reported as DFSP by pathologist	R0	37
18	C	F	DFSP	61	Trunk	On scar from nodular fasciitis and fibrous histiocytoma excision	1.2	2	VRAM flap	n/a	CD34+, MIB1 low	R0	12
19	A	F	DFSP	59	Inguinal	On scar from abscess excision	9.0	2	VRAM flap	n/a	CD34+	R0	12

*A* incisional or excisional biopsy at different hospitals with subsequent excision at our hospital, *B* biopsy and excision at our hospital, *C* re-excision at our hospital for recurrence after excision at different hospitals, *M* male, *F* female, *DFSP* dermatofibrosarcoma protuberans, *n/a* not applicable, *TRAM* transverse rectus abdominis myocutaneous, *LICAP* lateral intercostal artery perforator, *ALT* anterolateral thigh, *VRAM* vertical rectus abdominis myocutaneous

**Fig. 1:**
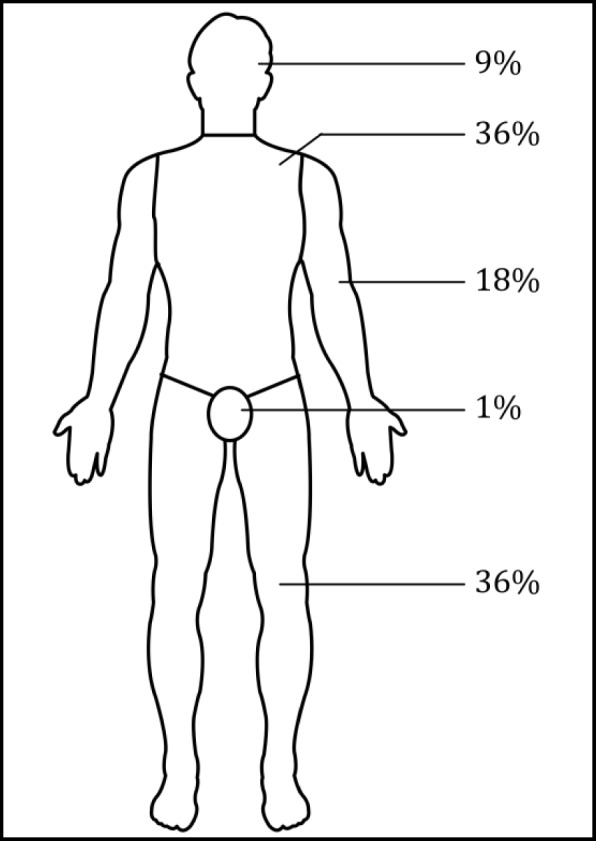
“Wallace rules of 9s” displaying the according body surface area percentages

In order to account for possibly disproportionate findings in tumor localization percentages, we applied the “Wallace rule of 9s” (Fig. 1) [10]. This rule is a rough estimate for body surface area in the clinical setting. A typical application of this estimate would be in burn victims for calculating the extent of skin damage. To adjust large body surface areas against smaller areas, we divided the percentage of tumors found in a certain anatomic area by the BSA percentage of this area as described by the “rule of 9s.” In doing so, we obtained a BSA adjusted numeral: BSA adjusted *=* $\frac{\%\mathrm{of}\ \mathrm{tumors}\ \mathrm{in}\ \mathrm{anatomic}\ \mathrm{area}}{\mathrm{BSA}\%\mathrm{of}\ \mathrm{anatomic}\ \mathrm{area}}$. Results were rounded to three significant figures.

The diagnosis of DFSP was established histopathologically by H&E staining and immunohistochemistry (CD34). In cases with equivocal features and/or cases of FSDFSP, fluorescent in situ hybridization (FISH) analysis to demonstrate the COL1A1-PDGFRB translocation was performed to confirm diagnosis.

Initial biopsies or close excisions were performed using no or narrow margins. Wide local excision was the mainstay of surgical treatment, which was performed with at least a 1–3 cm margin by removing the skin, subcutaneous tissue, and superficial fascia. In cases of non-complete or almost-complete resection or in cases treated with surgical resection at another hospital, additional wide excision was performed, including the surgical scar. The incision line was marked on the normal skin to obtain the planned distance from the tumor margin. Conventional surgery was adopted in areas in which wide excision would have been difficult to perform. A variety of surgical techniques were adopted for the most suitable wound closure, including primary intention and pedicled flaps, free flaps, or skin grafts, in cases of larger defects that did not permit primary closure.

Surgical margin assessment was conducted based on macroscopic and histopathological analyses. If the tumor size was reported in three dimensions (length, height, and width), the largest dimension was declared as tumor diameter. If a mass consisting of histologically grouped tumoral lesions was excised, a total tumor diameter was declared by adding diameters of all lesions. Four lateral margins (12, 3, 6, and 9 o’clock) and one deep margin were evaluated.

Follow-up was performed in checking medical records of our institution as well as conducting interviews. Patient follow-up for recurrence ranged from 5 to 175 months with a mean follow-up time of 78 months.

## Results

Our retrospective patient group includes 19 patients who were diagnosed with DFSP from 2002 to 2016. The clinicopathological parameters are illustrated in Table 1. Sex distribution was approximately equal with a total of 11 female and 8 male patients. Mean patient age at diagnosis was 53 years (range 33 to 89 years). Almost half of tumors (*n* = 10) were located on the trunk. Other anatomic locations included the lower extremities (*n* = 4), the upper extremities (*n* = 2), the groin (*n* = 2), and the head and neck area (*n* = 1). The results of the BSA adjustment to our patient group are depicted in Table 2. The most common location by BSA adjustment was the groin, followed by the trunk and the head. We applied the BSA adjustment formula to the findings of Kreicher et al. [2] (Table 3). The most common tumor localization adjusted to the respective body surface area percentage was the head (1.43) followed by the upper limbs (1.18) and the trunk. The least common location was the lower limbs (0.578). The groin area was not among the most common locations (1.02).

**Table 2:** BSA adjustment to patient group of our retrospective chart (*n* = 19)

	Number of patients	Percentage of total	BSA adjusted percentage
Trunk	10	52.6	1.46
Upper limb	2	10.5	0.583
Lower limb	4	20.1	0.558
Head	1	5.26	0.584
Groin	2	10.5	10.5

**Table 3:** BSA adjustment to patient group of Kreicher et al. [2]

	Number of patients	Percentage of total	BSA adjusted percentage
Trunk	2841	41.7	1.16
Upper limb	1442	21.2	1.18
Lower limb	1420	20.8	0.578
Head	880	12.9	1.43
Genitals	71	1.04	1.04
Other	163	2.4	–

Some patients with DFSP were referred to our clinic from primary clinics, for definite surgical treatment after incomplete excision or owing to recurrent lesions. Patients were categorized as primary versus recurrent cases. Τhe histopathological diagnosis before performing wide resection at our hospital was established as follows: 8 cases were diagnosed shortly after resection performed at another hospital (group A), 7 cases underwent incisional or excisional biopsy at our hospital (group B), and 4 cases were diagnosed at time of recurrence after surgery performed at another hospital (Group C).

Notably, 6 out of 19 patients recounted tumor growth on cutis that previously had been exposed to trauma, such as chronic irritation, laceration, or burns. Interestingly, DFSP grew on a former site of nodular fasciitis and former site of a fibrous histiocytoma in the same patient. The initial histology may have missed the correct diagnosis. Unfortunately, former histology specimens from the non-affiliated hospitals were not available for repeated histology.

Further resection was required in 15 patients who either were diagnosed with microscopic positive resection margins following primary excision at a different institution (*n* = 8) or underwent an incisional or excisional biopsy with R1 or R0 (close) margins at our institution (*n* = 7). All these patients received a complete and wide local tumor excision with microscopic negative resection margins (R0 resection).

Four patients (group C), who had undergone an excision with R0 situation at different institutions, suffered a tumor recurrence. Time to local recurrence in these four patients was 7, 31, 57, and 69 months (mean time to recurrence, 41 months; median time to recurrence, 44 months). They received a wide local excision with tumor-free margins.

Following prior incomplete excision (R1), a mean margin width of 1.50 cm was used to accomplish negative margins during repeat excision (group A). Negative surgical margins upon excisional or incisional biopsy (group B) were achieved by a mean surgical margin width of 2.04 cm. In patients who suffered from a recurrent tumor (group C), a mean margin width of 1.38 cm was sufficient to establish negative margins. Negative surgical margins were achieved in all patients (groups A, B, C) by a mean margin width of 1.67 cm.

**Fig. 2:**
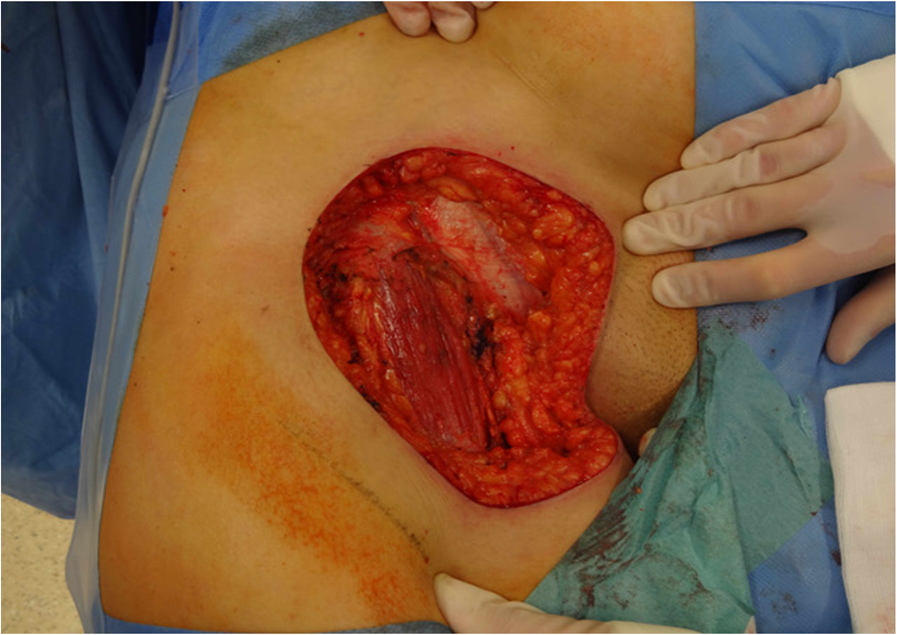
Inguinal soft tissue defect after resection of an inguinal DFSP with 2-cm safety margins in a 59-year-old woman

**Fig. 3:**
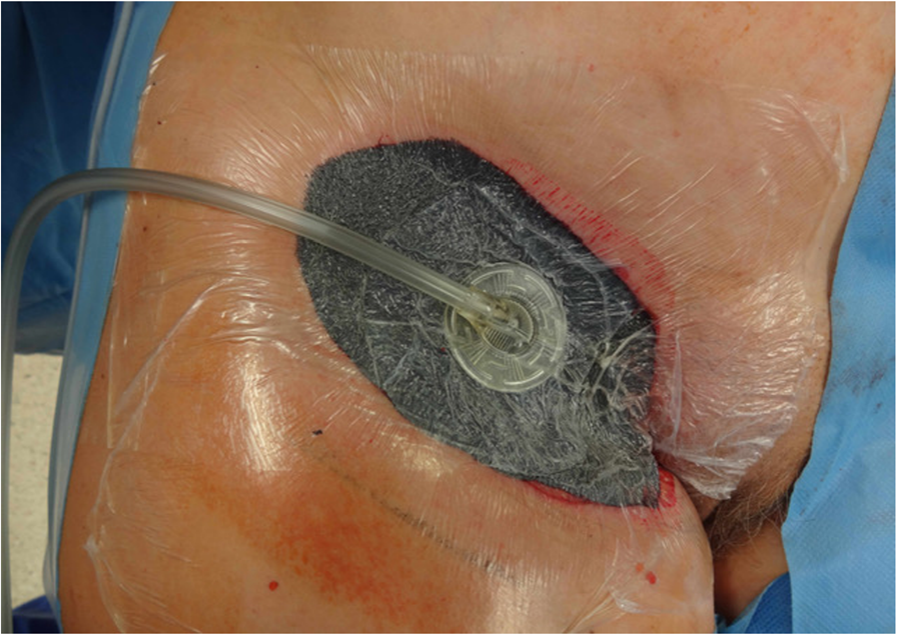
Defect covered with a negative pressure wound system

**Fig. 4:**
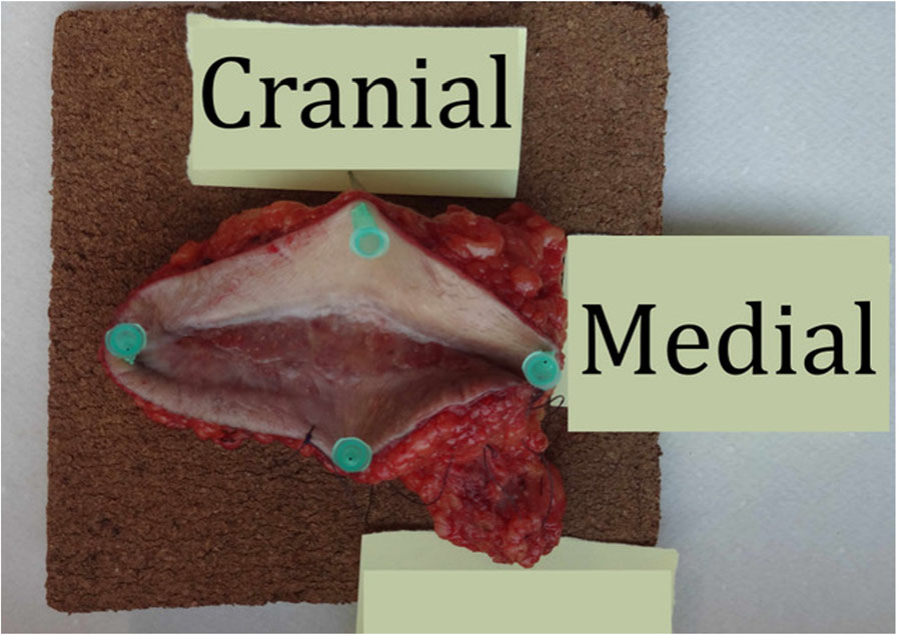
Inguinal specimen of DFSP (note the scar from prior R1 resection in the center of the specimen)

**Fig. 5:**
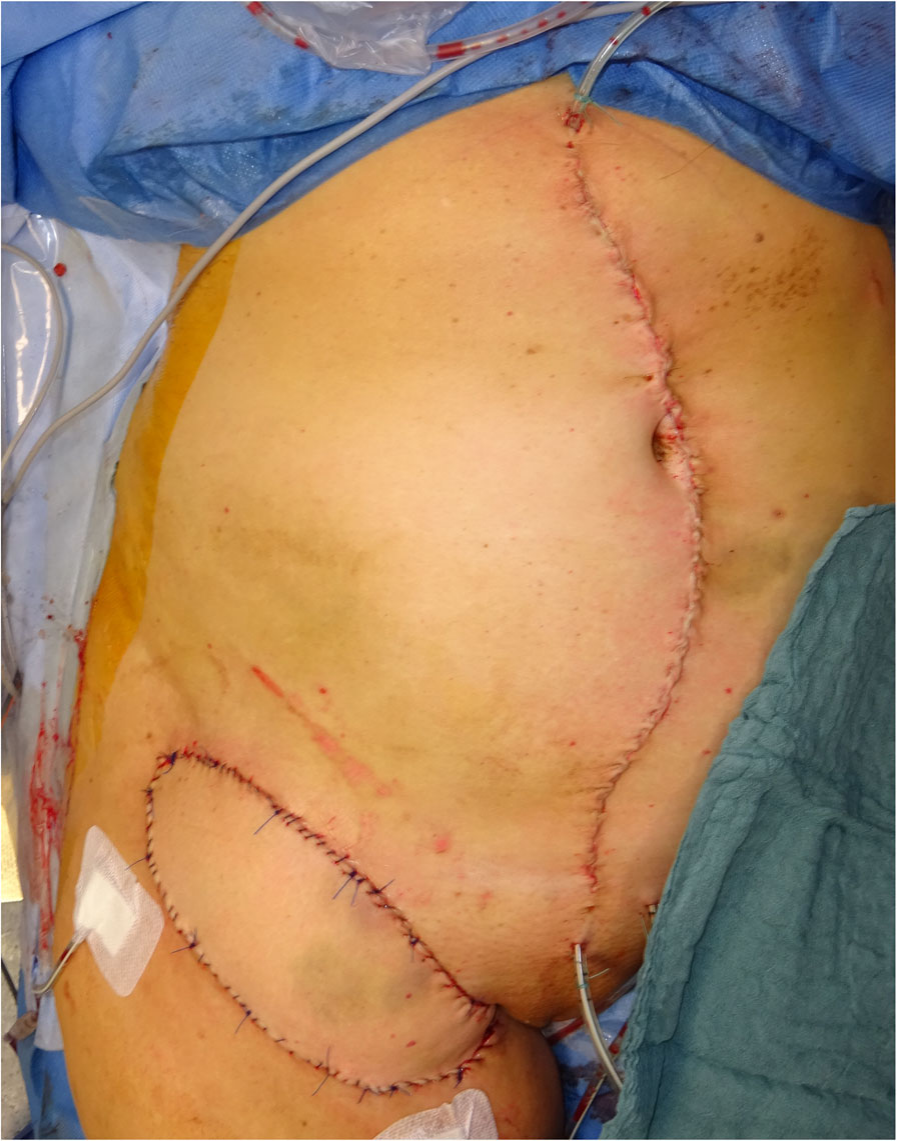
Plastic reconstruction with VRAM flap

Diameters of the resection specimens, including biopsies or primary close excisions, ranged from 0.3 to 9 cm with a median tumor size of 2.9 cm. Primary closure was performed in 8 patients, while the wound defects of the other 11 patients required plastic reconstruction (pedicled transverse rectus abdominis (TRAM) flap, *n* = 1; pedicled pectoralis flap, *n* = 1; free gracilis flap, *n* = 1; pedicled lateral intercostal artery perforator (LICAP) flap, *n* = 1; free anterolateral thigh (ALT) flap, *n* = 1; pedicled vertical rectus abdominis myocutaneous (VRAM) flap, *n* = 1; free (VRAM) flap, *n* = 1; with regional pedicled flaps, *n* = 2; skin grafts, *n* = 2) (Figs. 2, 3, 4 and 5).

Neoadjuvant therapy was administered in two patients. One of these patients had a high-grade fibrosarcomatous component and received radiation with 60 Gy as well as chemotherapy consisting of ifosfamide and doxorubicin. Excision was performed 2 months after radiation treatment. This patient developed impaired sensibility in the lower extremity due to radiation damage. The other patient received 400 mg imatinib mesylate daily for 6 months—without any long-term side effects—for size reduction before excision. The tumor exhibited a superficial cutaneous growth with a diameter of estimated 24 cm. Using imatinib, remarkable size reduction was achieved, given the final specimen of 6.5 cm as measured by the pathologist.

Following surgical excision, 2 out of 19 patients developed unfavorable sequelae, yielding a morbidity of 10.5%. According to the Clavien-Dindo classification, these sequelae may be ranked as grades I (seroma) and III-b (dog ear defects requiring surgical correction). There was no postoperative mortality.

The median follow-up was 84 months with a range of 6 to 175 months. No patient of our series developed any recurrent or metastatic DFSP. Two patients died of unrelated causes 6 and 58 months, respectively, after the diagnosis of DFSP.

## Discussion

DFSP is a rare mesenchymal malignant neoplasm. Its incidence in the USA ranges from 0.8 to 4.1 per million persons per year [1, 2]. Congenital cases have been described and incidence of DFSP increases until age 20 but not thereafter [6–8]. Accordingly, most cases of DFSP arise in the third decade of life. Mean patient age at diagnosis in our study group was slightly higher (53.2 years). In our study, sex distribution is equal, while some studies reported a slight preponderance of DFSP among women [2].

Grossly, DFSP shows a plaque-like thickening of the skin with violaceous, reddish-brown or normal skin color [11]. Raised nodules or a multinodular polyp growth represent the typical “protuberant” appearance when left untreated. Macroscopic lesion sizes vary from 0.5 to more than 12 cm [12]. Several morphologic variants have been described, such as a pigmented variant “Bednar tumor” [13]. Histologically, DFSP infiltrates the subcutis or underlying muscle with a characteristic honeycomb pattern. The tumor is characterized by storiform fascicles of uniform spindle cells that have little to no mitotic activity. Immunohistochemically, DFSP is CD34 positive and immunohistochemical staining for CD34 is often used for differential diagnosis from dermatofibroma [12–14].

Surgical excision remains the standard of therapy [15]. Main surgical treatment options consist of wide excision or Mohs micrographic surgery. There is no consensus on the exact safety margins in wide excision. According to Guidelines by the German Cancer Society and German Dermatologic Society, excision margins usually range from 1 to 5 cm. The authors advocate using margins of 2–3 cm as this seems “reasonable” [16]. Yet, no clear recommendations are available. A European consensus-based guideline recommends 3-cm margins [17]. While Woo et al. [18] propose margins of 1.5–2 cm, Archontaki et al. suggest using margins of at least 5 cm [19]. The Clinical Practice Guidelines in Oncology for Dermatofibrosarcoma Protuberans published by the National Comprehensive Cancer Network recommends 2- to 4-cm margins to investing fascia if wide local excision is applied. These Guidelines also emphasize the importance of excising the deep fascia to improve disease-free survival [15]. In our series, we achieved R0 margins using margin widths ranging from 0.5 to 3 cm. Our patient data shows that a mean margin width of 1.67 cm resulted in negative margins in all patients with a median recurrence-free survival of 84 months. Hence, using margins of 1–2 cm would seem feasible to achieve adequate control in primary tumor excision. However, some of these patients had undergone primary resection already. For this reason, the stated mean margin width of 1.67 cm must be calculated on top of prior resection widths. Negative margins upon primary biopsy were accomplished by a mean margin width of 2.04 cm in 7 patients.

Consequently, we would advocate using a margin width of at least 2 cm and scrupulous histopathological examination with re-excision upon close (R0) margins to achieve adequate results (Figs. 2, 3, 4, and 5). This finding concurs with the recommendations of Harati et al. [20] who also suggest 2–3-cm margins, as well as with the National Comprehensive Cancer Network (NCCN) Guidelines. Achieving adequate resection margins is key since recurrence is linked to positive margins [13, 21]. On the other hand, ample safety margins cause large defects that may require plastic reconstruction (*n* = 11). In these cases, the patients were treated with temporary V.A.C.® therapy (images 2–4) to receive reconstructive surgery in a second session.

Mohs micrographic surgery (MMS) is a technique that offers histopathologic control while ensuring minimal tissue defect [22]. While Lowe et al. [22] lean towards applying MMS rather than wide excision for DFSP, the NCCN guideline does not favor any particular approach. Some authors recommend MMS as it should provide sufficient histopathologic control during excision, ultimately resulting in lower recurrence compared to wide excision [17, 23]. However, micrographic procedures are lengthy and require the necessary equipment and trained staff. Both of which might not be available at every institution. Moreover, complex or large tumors may be resectable only or in combination with wide excision [16, 20–22]. MMS was not applied at our institution. In our study, however, 11 out of 19 patients required plastic reconstruction due to large defects caused by excision with safety margins of 1–3 cm. Hence, MMS may be considered in primary excision of tumors that are located at critical locations, such as the head or hands, to avoid unfavorable sequelae [22]. Notably, a study by Chen et al. found that MMS was economically equivocal to costs of wide excision in an operating room [24]. Furthermore, additional plastic reconstruction, which was frequently associated with wide excision treatment in our study, results in higher financial burden.

Molecular therapy using Imatinib mesylate, a tyrosine kinase inhibitor, targets the autocrine growth stimulus found in most DFSP. In 2006, Imatinib was approved by the European Medicines Agency (EMA) for metastatic disease, unresectable recurrent, or unresectable primary tumors [17]. In the USA, the Food and Drug Administration (FDA) has approved the use of Imatinib mesylate in 2006 in adult patients with unresectable, metastatic, or recurrent DFSP [15]. One patient of our study group was treated with imatinib for size reduction in advance of surgical excision. We assume this initial size reduction to be beneficial since the patient did not suffer any tumor recurrence following excision. Bednar tumor or tumors lacking a t(17;22) translocation are thought to not respond to imatinib treatment [13, 25, 26].

According to European guidelines, radiotherapy may be used as neoadjuvant therapy for primary inoperable tumors or recurrent disease [17]. It may be used as adjuvant therapy following surgery with positive resection margins [17]. Chen et al., who conducted a meta-analysis of the efficacy of adjuvant radiotherapy, even suggest using adjuvant radiotherapy if negative margins have been achieved [27]. However, their analysis did not include any randomized controlled trials and included a total of 12 studies. An effective chemotherapy is currently unavailable [13, 17]. Yet, one patient in our group suffered from DFSP with a high-grade fibrosarcomatous component. For this reason, the patient received doxorubicin and ifosfamide as neoadjuvant therapy. As to this date, the patient has not suffered from any recurrence. Nevertheless, we cannot draw any conclusions regarding adjuvant therapy due to small sample size.

Most common locations for DFSP as reported in literature are the trunk followed by the extremities [2, 28–30]. Interestingly, there are several case reports that describe DFSP arising from a preexisting trauma to the skin, such as vaccination, tattoos, radiation, or burns [31–35]. However, the torso also represents a large body surface area (36%). Above all, no relationship between tumor localization and tumor pathogenesis has been elucidated yet for DFSP. In our case series, we discovered that 9 out of 19 patients (47.4%) had a DFSP arising from an anatomical location other than the trunk. Six patients recounted prior trauma to the part of the skin that would transform into DFSP eventually.

We investigated if certain anatomic regions are more likely to foster growth of DFSP than other areas of the body. In order to account for the different sizes of these areas, we compared tumor site prevalence with body surface area percentages. However, our study group included a small number of patients. This small number caused statistically questionable results (Table 2) as evident in the irregularly high numeral for the groin area (10.5) versus the remaining areas (ranging from 0.558 to 1.46). Therefore, we intended to apply the BSA adjustment to findings of a study that comprised a large number of patients. Kreicher et al. [2] conducted a large population-based study on the incidence of DFSP in the USA, including 6817 patients. We applied the BSA adjustment formula to their respective tumor localization results (Table 3) and found that the trunk was not the most common location. However, the “rule of 9s” was designed as a rule that is easy to memorize. Therefore, it quotes integer percentages and multiples of the number 9. When used in our calculations, these approximate numbers generate very approximate results. These results are very likely statistically insignificant. Additionally, the “rule of 9s” holds mostly true for the average adult but not for other patient populations [36]. Nevertheless, we hypothesize that the skin tissue found on the trunk is not more likely to promote growth of DFSP than the skin of other body areas. We suggest to abstain from citing the trunk as the most common tumor localization of DFSP. Instead, we propose to describe the tumor as a ubiquitous soft tissue sarcoma with a tendency to grow in skin that has been exposed to prior trauma.

Patients diagnosed with DFSP who underwent a complete excision have an excellent prognosis with a 5-year-survival rate of 99% [8]. Median time to local recurrence is about 3 years [37]. Follow-up in 6–12-month intervals is recommended as DFSP has a rather high rate of recurrence [15]. The follow-up should be continued well beyond 5 years since recurrences after this period are not uncommon in DFSP [38]. The median follow-up in this retrospective study was 84 months (7 years) with all patients being disease free as recorded until July 2017. Two patients out of a total of 4 patients with tumor recurrence following incomplete excision at away institutions were noted well beyond an interval of 5 years. Therefore, we concur that follow-up should be continued after 5 years.

## Conclusion

In summary, DFSP is a relatively rare, ubiquitous tumor of the soft tissue with challenging characteristics. We report nineteen cases of DFSP treated at our institution. All patients received wide excision with safety margins of 0.5–3 cm. In our experience, we would advocate using safety margins of at least 2 cm. Unfortunately, wide excision with safety margins often leads to large soft tissue defects that ultimately require plastic reconstruction. Alternatives like Mohs micrographic surgery that could result in lower need for reconstructive surgery may be considered in the future to avoid these unfavorable sequelae.

## Abbreviations

ALT: Anterolateral thigh
BSA: Body surface area
DFSP: Dermatofibrosarcoma protuberans
EMA: European Medicines Agency
FDA: Food and Drug Administration
FISH: Fluorescent in situ hybridization
FS-DFSP: Fibrosarcomatous dermatofibrosarcoma protuberans
LICAP: Lateral intercostal artery perforator
MMS: Mohs micrographic surgery
NCCN: National Comprehensive Cancer Network
TRAM: Transverse rectus abdominis
VRAM: Vertical rectus abdominis myocutaneous
